# Gene-centric association analysis for the correlation between the guanine-cytosine content levels and temperature range conditions of prokaryotic species

**DOI:** 10.1186/1471-2105-11-S11-S7

**Published:** 2010-12-14

**Authors:** Hao Zheng, Hongwei Wu

**Affiliations:** 1 School of Electrical and Computer Engineering, Georgia Institute of Technology, USA

## Abstract

**Background:**

The environment has been playing an instrumental role in shaping and maintaining the morphological, physiological and biochemical diversities of prokaryotes. It has been debatable whether the whole-genome Guanine-Cytosine (GC) content levels of prokaryotic organisms are correlated with their optimal growth temperatures. Since the GC content is variable within a genome, we here focus on the correlation between the genic GC content levels and the temperature range conditions of prokaryotic organisms.

**Results:**

The GC content levels in the coding regions of four genes were consistently identified as correlated with the temperature range condition when the association analysis was applied to (*i*) the 722 mesophilic and 93 thermophilic/hyperthermophilic organisms regardless of their phylogeny, oxygen requirement, salinity, or habitat conditions, and (*ii*) partial lists of organisms when organisms with certain phylogeny, oxygen requirement, salinity or habitat conditions were excluded. These four genes are K01251 (adenosylhomocysteinase), K03724 (DNA repair and recombination proteins), K07588 (LAO/AO transport system kinase), and K09122 (hypothetical protein).

To further validate the identified correlation relationships, we examined to what extent the temperature range condition of an organism can be predicted based on the GC content levels in the coding regions of the selected genes. The 84.52% accuracy for the complete genomes, the 84.09% accuracy for the in-progress genomes, and 82.70% accuracy for the metagenomes, especially when being compared to the 50% accuracy rendered by random guessing, suggested that the temperature range condition of a prokaryotic organism can generally be predicted based on the GC content levels of the selected genomic regions.

**Conclusions:**

The results rendered by various statistical tests and prediction tests indicated that the GC content levels of the coding/non-coding regions of certain genes are highly likely to be correlated with the temperature range conditions of prokaryotic organisms. Therefore, it is promising to carry out “reverse ecology” and to complete the ecological characterizations of prokaryotic organisms, i.e., to infer their temperature range conditions based on the GC content levels of certain genomic regions.

## Background

There are countless ways in which prokaryotes influence our daily life. For example, they mediate the chemical cycles that convert key elements of life into biologically accessible forms; they make certain nutrients/metals/vitamins available to their biological hosts; and they can also be used to breakdown hydrocarbons and treat crude oil leakages. On the other hand, the environment has undoubtedly left footprints on the morphological, physiological and biochemical diversities of prokaryotes during the evolution. The close interactions between prokaryotes and the environment, especially driven by horizontal gene transfer and homologous recombination, have made prokaryotes the most genetically diverse superkingdoms of life [1].

Temperature is one of the elements characterizing the ecological contexts of prokaryotic organisms. The National Center for Biotechnology Information (NCBI) Microbial Genome Project Database (http://www.ncbi.nlm.nih.gov/genomes/lproks.cgi) uses five terms to categorize the temperature range an organism grows at, where cryophilic refers to -30° to -2°C, psychrophilic refers to -1° to +10°C, mesophilic refers to +11° to +45°C, thermophilic refers to +46 to +75°C, hyperthermophilic refers to above +75°C, and organisms that live at ranges that overlap with more than one category are labeled as the one corresponding to the largest overlap. It has been reported that temperature can possibly influence the ecological, physiological and genomic properties of a prokaryotic organism in multiple aspects. For instance, at the population-level, temperature was shown to have caused compositional and functional shifts in microbial communities [2]; at the cellular level, temperature was shown to have a significant effect on a variety of growth parameters (e.g., optical density, viable cell numbers, and cell dry mass) [3], structure and ion permeability of cell membranes [4], affinity for substrates (e.g., glycerol and nitrate) [5,6], circadian rhythms [7], and virulence functions [8]; and, at the molecular level, temperature was shown to be correlated with the nucleotide content, codon usage and amino acid composition [9-12], structure/function/stability of proteins [13,14], topological properties of metabolic networks [15], expression of certain genes (e.g., heat-and cold-shock response genes) [16-18], etc.

Guanine-Cytosine (GC) content is one of the genomic traits that have been hypothesized to be correlated with the temperature condition of an organism. Since the GC pair is bound by three hydrogen bonds while the adenine-thymine (AT) pair is bound by two hydrogen bonds, it has long been expected that organisms growing at higher temperature would have a higher proportion of GC than AT pairs. However, it remains ambiguous whether the whole-genome GC content level of an organism is correlated with its temperature condition. Analysis on 368 bacterial species seemed to have confirmed the existence of the positive correlation between the whole-genome GC content level and the optimal growth temperature of prokaryotes [19]. However, later analysis indicated that the sample size, presence of outliers, as well as some other factors that may affect whole-genome GC content levels (e.g., mutational bias, genome size, oxygen requirement, nitrogen utilization, habitat, salinity and alkalinity) could potentially introduce bias towards the association analysis and lead to questionable conclusions [20-22]. Actually, organisms living at high temperature have mechanisms other than increasing GC content, such as thermophile-specific enzymes (e.g., reverse gyrase) [23] or certain dinucleotides that may contribute to thermostability [24], to maintain the double stranded structure of the DNA.

**Figure 1:**
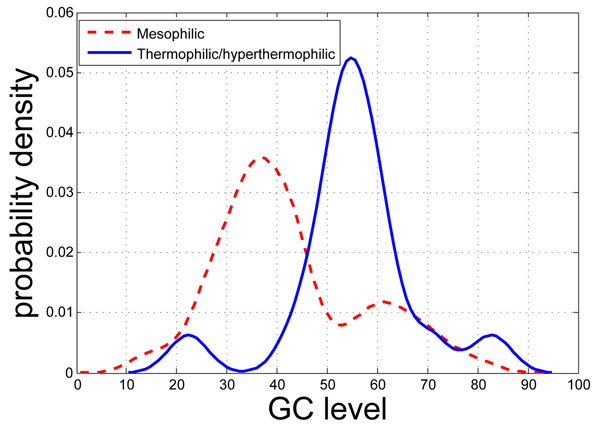
The distribution of the GC content level of the non-coding region surrounding the menB gene (K01661: naphthoate synthase) for mesophilic (dashed, red) and thermophilic/hyperthermophilic organisms (solid, blue).

It is worthy of notice that the GC content can be substantially variable within the same genome. For instance, the GC content in coding regions is often higher than that of the whole genome [25]. And, if a DNA fragment is obtained via a recent horizontal gene transfer event, its GC content tends to exhibit different variation patterns from the native parts of the genome. Despite the lack of obvious correlation between the whole-genome GC content level and the optimal growth temperature, studies have shown that the GC content levels of certain genes (e.g., ribosomal and transfer RNA genes) are significantly correlated with the optimal growth temperatures [26]. Also, as shown in Fig. 1, the non-coding region surrounding the gene menB (naphthoate synthase) can be drastically different for mesophilic and thermophilic/hyperthermophilic organisms. Inspired by these preliminary investigations, we here focus on the correlation relationships between the *genic* GC content levels and the temperature range conditions of prokaryotic organisms.

## Data sets

### Genomes

Among the 895 complete prokaryotic genomes available from the Kyoto Encyclopedia of Genes and Genomes (KEGG) (http://www.genome.jp/kegg/, July 2009 release), 829 genomes are accompanied with characterizations of temperature range conditions in NCBI’s Microbial Genome Project Database (http://www.ncbi.nlm.nih.gov/genomes/lproks.cgi). The temperature range of a prokaryotic organism is characterized by one of the five terms – cryophilic, psychrophilic, mesophilic, thermophilic and hyperthermophilic; however, the organisms are unevenly distributed under these five temperature range conditions, with the majority falling into the category mesophilic (Table 1). To maximally take advantage of the provided information regarding organisms’ temperature range conditions as well as to obtain statistically significant results, we excluded the cryophilic and psychrophilic categories from further analysis, merged 57 thermophilic and 36 hyperthermophilic organisms into the same category (labeled as thermo/hyperthermo-philic), and then focused on the 722 mesophilic versus 93 thermo/hyperthermo-philic organisms.

**Table 1:** Number of organisms under various temperature range, oxygen requirement, habitat and salinity conditions, where the number in the parentheses is the number of organisms for a specific combination of the temperature range condition and a condition of some other environmental factor.

Condition	#Org	Habitat	Oxygen	Salinity
Psychrophilic	14	Aquatic(5)	Facultative(7)	Mesophilic(2)
		Specialized(4)	Aerobic(3)	-
		-	-	-

Mesophilic	722	Host-associated(284)	Facultative(282)	Non-halophilic(149)
		Multiple(242)	Aerobic(244)	Mesophilic(10)
		Aquatic(111)	Anaerobic(99)	Moderate halophilic(9)

Thermophilic	57	Specialized(36)	Anaerobic(26)	Non-halophilic(4)
		Aquatic(10)	Aerobic(13)	Moderate halophilic(2)
		Multiple(6)	Facultative(11)	Mesophilic(1)

Hyperthermophilic	36	Specialized(21)	Anaerobic(22)	Non-halophilic(3)
		Aquatic(12)	Aerobic(8)	Moderate halophilic(3)
		-	-	Mesophilic(2)

### Genic GC content

Among the 2,798,133 genes of the 815 organisms being studied, 1,210,908 (~43.3%) genes are assigned with KEGG Orthology (KO) IDs, and a total number of 6,026 KO groups are being covered. We considered genes with the same KO IDs as orthologous, so that they can be used to estimate the distribution of the GC content level surrounding each gene (corresponding to a unique KO ID) in various genomes. For each gene, we obtained the GC content levels of both the coding and surrounding non-coding regions, where the non-coding region starts from the end of the previous gene till the start of the next gene with the coding region of the current gene being excluded. As the coding and non-coding regions were treated independently, we used *genomic region* to refer to either of them in the rest of the paper.

## Methods

Our association analysis consisted of two steps – statistical tests and prediction tests. The Kolmogorov-Smirnov (KS) statistical tests were first carried out to identify those genomic regions of which the distribution patterns of the GC content levels are different for organisms of different temperature range conditions but are irrelevant to the phylogenetic distribution of the organisms or the distribution of other environmental factors. The support vector machine (SVM)-based prediction tests were then carried out to determine whether the temperature range condition of a prokaryotic organism can be inferred from the GC content levels of the genomic regions selected via the statistical tests.

### Statistical tests

#### KS tests on the complete list of organisms

The GC content estimates for each genomic region can be organized into two groups – one consisting of those obtained from the 722 mesophilic organisms and the other consisting of those obtained from the 93 thermo/hyperthermo-philic organisms. The KS test was conducted to determine whether these GC content levels are distributed differently for these two groups of organisms of different temperature range conditions. Specifically, the GC content of a genomic region was considered as potentially correlated with the temperature range condition if its corresponding *p*-value was less than 0.001. In order to increase the stability of the identified correlation relationships, we also adopted the bootstrap strategy [27] to repeatedly perform the KS test on 90% of the randomly selected mesophilic organisms versus all the thermo/hyperthermo-philic organisms for 200 iterations. Those genomic regions that were consistently selected in all the 200 KS tests, denoted as {*g_complete_*}, were then taken for further analysis.

#### KS tests on partial lists of organisms with each individual phylum being excluded in turn

**Figure 2:**
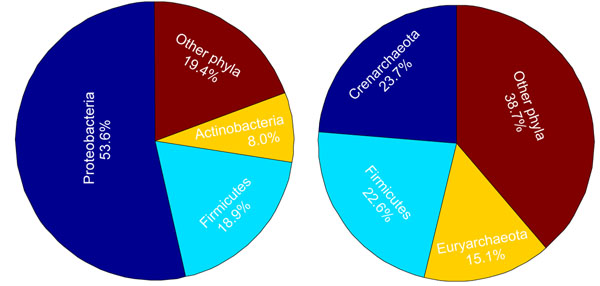
The distribution of the mesophilic organisms (left) and the distribution of the thermo/hyperthermo-philic organisms (right) among various phylogenetic groups.

The distribution of the mesophilic organisms in various phylogenetic groups are different from that of the thermo/hyperthermo-philic organisms. Observe from Fig. 2 that mesophilic organisms mainly fall into the *Proteobacteria, Firmicutes* and *Actinobacteria* phyla; whereas, thermo/hyperthermo-philic organisms mainly fall into the *Crenarchaeota, Firmicutes* and *Euryarchaeota* phyla. This may prompt one to ask whether the difference in the phylogenetic distributions of these organisms also contributes to the difference in the distributions of the GC content levels of the genomic regions in {*g_complete_*}.

To examine whether the distribution of the GC content level is correlated with the phylogenetic origin of an organism and whether the temperature range condition and the phylogenetic origin are coupled in being correlated with the distribution of GC content levels, we should ideally perform the multi-variate ANOVA [28] or chi-square test [29]. However, the multi-variate ANOVA assumes that all sample populations under different conditions are normally distributed and have equal variance, which is not necessarily satisfied for our small data set. The chi-square test requires that predictor variables are categorical, which is not satisfied in our case since the predictor variable, the GC content level, is continuous. We therefore turned to the approach of leaving an individual phylum out and performing the KS test on the remaining organisms. The KS test on the partial list of organisms was performed 26 times with one phylum being excluded for each time. The genomic regions in {*g_complete_*} that were also selected when a phylum was excluded, {*g*_–_*_phylum_*}, can be viewed as correlated with the temperature range condition but irrelevant to the distribution of the phylum being excluded. And, the genomic regions that were shared by all {*g_–phylum_*}’s can then be viewed as correlated with the temperature range condition but irrelevant to the distribution in any phylum.

#### KS tests on partial lists of organisms with particular oxygen requirement, habitat or salinity conditions being excluded

Various physiological features, including the gram stain, shape, arrangement, endospores, motility, salinity, oxygen requirement, habitat, organisms that the prokaryote is pathogenic in, and the related disease, are together with the temperature range conditions provided in NCBI’s Microbial Genome Project Database for each prokaryotic genome. Besides the temperature range condition, we were also interested in the features regarding the salinity, oxygen requirement, and habitat, because (*i*) these features characterize the basic environment that an organism prefers to or has been found to live in, and (*ii*) these features can be specified by using controlled vocabularies.

Observe from Table 1 that the distribution of the mesophilic organisms under various oxygen requirement, habitat and salinity conditions is different than that of the thermo/hyperthermo-philic organisms. To investigate the possible interactions among different environmental factors in their being correlated with the GC content levels, we adopted similar strategy as for the phylogenetic factors, i.e., to exclude organisms of a particular oxygen requirement/salinity/habitat condition and then conduct the KS tests on the remaining organisms. This procedure was repeated for all the oxygen requirement, salinity, and habitat conditions. The genomic regions in {*g_complete_*} that were also selected when a particular environmental condition was excluded is denoted as , and can be considered as correlated with the temperature range condition but irrelevant to the distribution in the environmental condition being excluded. And, the genomic regions that are shared by all ’s can then be considered as correlated with the temperature range condition but irrelevant to the distribution in any of the other three environmental factors being considered.

#### Statistical tests on the Genomes OnLine Database (GOLD)

**Figure 3:**
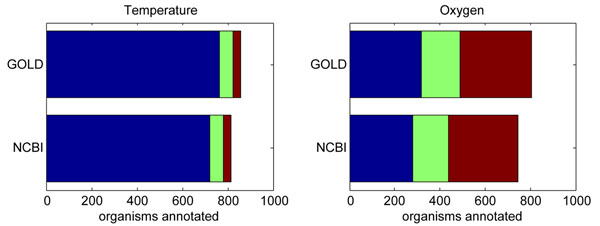
Comparisons of environmental annotations in the NCBI and GOLD databases for the 895 complete prokaryotic genomes. For temperature range, the number of annotated organisms for three dominant conditions (mesophilic, thermophilic and hyperthermophilic) are shown in blue, green and brown, respectively. For oxygen requirement, the number of annotated organisms for three dominant conditions (aerobic, anaerobic and facultative) are shown in blue, green and brown, respectively.

The reported association analysis on the genomic and environmental traits might be prone to the annotation errors at both the genomic and environmental sides. At the genomic side, since the annotations in the KEGG database involve both computation-based and manual curation [30], our estimates for the GC content levels in the coding/non-coding regions based on the KO annotations can be trusted. At the environmental side, however, there may exist errors in the NCBI’s Microbial Genome Project Database. For instance, it has been shown that the oxygen requirement annotations and/or habitat annotations of some prokaryotic organisms are problematic [31,32]. To examine to what extent the error in the environmental annotations may have affected our association analysis, we used the environmental annotations provided by the Genomes OnLine Database (GOLD) [33] and performed similar statistical tests. The GOLD database characterizes the ecological context of each organisms in 17 aspects, including the temperature range, oxygen requirement, salinity and habitat conditions. However, these two databases are not always consistent. For instance, the salinity condition of the species *Rhodobacter sphaeroides* KD131 is annotated as halophilic in the GOLD database but non-halophilic in the NCBI’s Microbial Genome Project Database; and the habitat condition of the species *Candidatus Methanoregula boonei* is annotated as fresh water in the GOLD database but terrestrial in the NCBI’s Microbial Genome Project Database. A systematic comparison of the environmental annotations of the two databases revealed that ~11.51% of the organisms have different temperature range annotations, ~15.6% of the organisms have different oxygen requirement annotations (see Fig. 3), and for the salinity and habitat conditions different vocabularies are adopted. By using the environmental annotations in the GOLD database, we performed all the above-mentioned KS tests, including those based on the complete list of the genomes as well as those based on the partial lists of the genomes with certain phylogenetic/oxygen requirement/habitat/salinity categories being excluded.

### Prediction tests

From the statistical point of view, the significance of the correlation relationships between the temperature range condition and the GC content levels of the selected genomic regions can be measured by the *p*-values derived from the KS tests. On the other hand, if the temperature range condition of an organism is predictable based on the GC content levels in the selected genomic regions, then the correlation relationships between the two traits can be further justified.

For the prediction test, each organism was represented by a feature vector consisting of the GC content levels of the selected genomic regions. A cross validation procedure [34] was applied to estimate the prediction accuracy. That is, the collection of the mesophilic genomes and the collection of the thermo/hyperthermo-philic genomes were each randomly partitioned into two portions. The portions with 10% of the genomes from both collections were used for testing to estimate the classification accuracy, while the other portions with 90% of the genomes were used for building a SVM-based classifier. This partition-training-and-testing procedure was repeated for 500 times to obtain the average prediction accuracy. The estimate for the prediction accuracy was benchmarked against the prediction accuracy rendered by random guessing. To facilitate such comparisons, we enforced that for each cross validation experiment the same number of organisms (93 vs. 93) for both temperature range conditions were used so that the accuracy rate rendered by random guessing is 50%. Note that the 500 repeats of the cross validation procedure could allow us to sample a variety of combinations of mesophilic organisms so that the estimate of the prediction accuracy was unbiased to particular sub-collections of mesophilic organisms.

To test the generalizability of the identified correlation relationships, we also applied the SVM-based classifier to predicting the temperature range conditions of 17 mesophilic and 17 thermo/hyperthermo-philic in-progress genomes in NCBI’s Microbial Genome Project Database (see Table S-2 of the supplementary material), as well as 29 mesophilic and 29 thermo/hyperthermo-philic metagenomes in the Integrated Microbial Genomes (IMG/M) system [35] (see Table S-4 of the supplementary material). The prediction accuracy for these in-progress genomes and metagenomes was averaged cross the 500 SVM classifiers, each of which was derived from the training phase of the above-mentioned cross validation procedure.

## Results and discussion

We here discuss the results of the statistical and prediction tests.

### Genes selected based on the complete list of organisms, {*g_complete_*}

A collection of 413 genomic regions (including the coding and non-coding regions of 80 genes, the coding regions of 197 genes, and the non-coding regions of 56 genes) were consistently detected in all the 200 bootstrap KS tests and were included into {*g_complete_*}. To find out the biological implications underlying these 413 genomic regions, we performed enrichment analysis to determine which KEGG pathways are over-represented by the pertinent genes of these genomic regions. The enrichment factor of a KEGG pathway is defined as the ratio of the percentage of the genes involved in this pathway among the genes in {*g_complete_*} to the percentage of the genes involved in the same pathway among all the genes being considered. And, a KEGG pathway was considered to be enriched if its enrichment factor is greater than 1. Here we summarize the KEGG pathways with the 10 largest enrichment factors in Table 2.

**Table 2:** Top 10 KEGG pathways enriched by genes of {*g_complete_*}.

Pathway ID	Specific Level Description	General Level Description
ko02040	Flagellar assembly	Cell Motility
ko02030	Bacterial chemotaxis	Cell Motility
ko00471	D-Glutamine and D-glutamate metabolism	Metabolism of Other Amino Acids
ko00643	Styrene degradation	Xenobiotics Biodegradation and Metabolism
ko00720	Reductive carboxylate cycle (CO2 fixation)	Energy Metabolism
ko00523	Polyketide sugar unit biosynthesis	Biosynthesis of Polyketides and Nonribosomal Peptides
ko00130	Ubiquinone and menaquinone biosynthesis	Metabolism of Cofactors and Vitamins
ko00521	Streptomycin biosynthesis	Biosynthesis of Secondary Metabolites
ko00271	Methionine metabolism	Amino Acid Metabolism
ko00730	Thiamine metabolism	Metabolism of Cofactors and Vitamins

It can been seem from Table 2 that one of the most enriched KEGG pathways is cell motility. It has been reported that the temperature affects bacterial movements in both chemotaxis (ko02030) and flagellar assembly (ko02040). For instance, flagella synthesis in *Escherichia coli, Proteus sp.* and *Salmonella sp.* are all inhibited at higher incubation temperature [36]. And, as for chemotaxis behavior, most motors of a motile bacterial cell spin exclusively clockwise at very low temperatures so that the cell tumbles more frequently; and, when the environment temperature is incremented, the cell usually show some increase in average translational velocity [37,38]. Note that styrene degradation (ko00643), ubiquinone and menaquinone biosynthesis (ko00130), and streptomycin biosynthesis (ko00521) are also among the top 10 enriched KEGG pathways, suggesting that these pathways are also subject to thermal influence. In accordance with these computation-based findings are the following biological experiment-based findings that are documented in various publications: (*i*) An increase of the temperature from 32°C to 40°C effectively decreases the styrene degradation rate of *Rhodococcus pyridinovorans* PYJ-1 to ~66% of the optimum value [39]. (*ii*) Ubiquinone-8 formation in *Escherichia coliK-12* strain AB 2847 was greatly affected by temperature variations – the rate of converting 2-octaprenyl phenol to ubiquinone-8 reached the maximum at 32°C, while virtually no reaction at all occurred at a temperature of 0°C [40]. And, (*iii*) Production of streptomycin in *Streptomyces griseus* ATCC 12475 was suppressed at elevated growth temperature, and even failed at 34°C or above [41]. These findings demonstrated that our computation-based analysis on the correlation between genomic and ecological traits may guide experimental investigations on the mechanisms of prokaryotic organisms adapting to environmental changes.

When based on the GC content levels of the genomic regions in {*g_complete_*}, the prediction accuracy, averaged over the 500 cross-validation experiments, was 92.45% for the complete genomes, and was 92.05% for the in-progress genomes. Note that both of the accuracy rates were significantly higher than the 50% accuracy yielded by random guessing.

### Genomic regions shared by all experiments on the partial lists of organisms

There were four genomic regions in {*g_complete_*} consistently selected during all the KS tests on the partial lists of organisms with particular phylogenetic/oxygen requirement/habitat/salinity categories being excluded. Table 3 summarizes the mean and standard deviation of the GC content level in each of these four genomic regions for mesophilic as well as thermo/hyperthermo-philic organisms. Observe that the GC content levels in these four regions tend to be higher in mesophilic than thermo/hyperthermo-philic organisms, which confirms the hypothesis that prokaryotic organisms living in high temperature range conditions do not necessarily rely on high GC contents to cope with their environment [23,24].

**Table 3:** The mean and standard deviation (std) of the GC content level in the four consistently selected genomic regions for mesophilic and thermo/hyperthermo-philic organisms, the genes corresponding to these four genomic regions and their functional annotations.

KO Group	Function Description	mean±std	

		mesophilic	thermo/hyperthermo-philic
**K01251**	Adenosylhomocysteinase	57.35 ± 9.13%	48.61 ± 10.47%
**K03724**	DNA repair and recombination proteins	62.17 ± 11.85%	47.90 ± 12.99%
**K07588**	LAO/AO transport system kinase	62.03 ± 9.96%	49.10 ± 13.40%
**K09122**	Poorly Characterized	66.04 ± 10.26%	45.43 ± 11.03%

These four genomic regions correspond to the coding regions of K01251 (adenosylhomocysteinase), K03724 (DNA repair and recombination proteins), K07588 (LAO/AO transport system kinase), and K09122 (hypothetical protein), and are interpreted as temperature-correlated but irrelevant to the distribution in various oxygen requirement, salinity, habitat and phylogenetic groups. Some of these computation-based findings can be further supported by experiment-based findings. For instance, K01251 reflects a common adaptation mechanism at high temperatures, because all the residues forming the network of aromatic and hydrophobic contacts and the residues potentially involved in cofactor binding are fairly well conserved among the hyperthermophilic but not in the mesophilic organisms [42]. Hyperthermophilic organisms may require DNA damage repair (K03724) to be unusually effective to cope with the destabilization of the DNA at high temperatures [43]. And, micro-array experiments on the *Escherichia coli* strains (record GDS1848) that had been entrained in the high temperature conditions (41.5°C degrees) also showed that the expressions of K03724 (lhr) and K07588 (argK) were significantly different than those of the wild strain that lives at 37°C degrees [44,45].

The phylogenetic trees of these four genes were built by using ClustalW2 [46] and Phylip [47], and are shown in the supplementary material. Based on these trees, we calculated the evolutionary distances between organisms of the same temperature range (intra-temperature) and the evolutionary distances between organisms of different temperature range conditions (inter-temperature). On average, the inter-temperature distances are 1.12, 2.29, 1.54, and 2.13; and, the intra-temperature distances are 0.79, 1.80, 1.16 and 1.48 for K01251, K03724, K07588, K0912, respectively. Therefore, in terms of the evolutionary trajectories of these four genes, organisms of the same temperature range conditions are closer to each other than they are to organisms of different temperature range conditions. When comparing the number of neighboring species with the same temperature range conditions in the phylogenetic trees built from the four genes against that based on the 16S rRNA genes (Table 4), we did not observe statistically significant differences, indicating that phylogenetically close species tend to inhabit the same temperature range conditions.

**Table 4:** Number of neighboring organisms with the same temperature range conditions in the phylogenetic trees built from the four genes and the 16S rRNA genes. The number in parentheses is the number of organisms with the corresponding gene present.

Temperature	group 1 (412)		group 2 (288)		group 3 (223)		group 4 (107)	

	K01251	16S	K03724	16S	K07588	16S	K09122	16S
**Mesophilic**	323	318	234	230	158	161	52	53
**Thermo/hyperthermo-philic**	57	59	34	33	35	39	43	45

When based on these four genomic regions, the prediction accuracy, averaged on the 500 cross-validation experiments, was 84.52% for the complete genomes (84.46% for mesophiles, 85.09% for thermo/hyperthermo-philes), 84.09% for the in-progress genomes (83.05% for mesophiles, 85.13% for thermo/hyperthermo-philes), and 82.70% for the metagenomes (79.31% for mesophiles, 86.21% for thermo/hyperthermo-philes), respectively, all of which were much higher than the 50% accuracy rendered by random guessing. It should be noted that with less than 1% of the elements in {*g_complete_*} as the features, the prediction accuracy only degraded 8.6% when compared to that rendered by the entire {*g_complete_*}, suggesting that these four genomic regions are predominantly correlated with the temperature range condition.

### Association analysis based on the environmental annotations in GOLD

When the KS tests were conducted on the mesophilic vs. thermo/hyperthermo-philic organisms based on the temperature range annotations in the GOLD database, eight genomic regions were consistently selected out of the tests on the complete list of organisms and on the partial lists of organisms with particular phylogenetic/oxygen requirement/habitat/salinity categories being excluded. These genomic regions include the coding regions of K01251 (adenosylhomocysteinase), K03724 (DNA repair and recombination proteins), K07588 (LAO/AO transport system kinase), K08289 (purine metabolism), and K09122 (hypothetical protein), and the non-coding regions of K00261 (glutamate dehydrogenase), K03809 (Trp repressor binding protein), and K07008 (currently unclassified). Note that the coding regions of K01251, K03724, K07588 and K09122 were the genomic regions consistently selected when the KS tests were based on the temperature range annotations in the NCBI’s Microbial Genome Project Database. That is, despite the 11.51% inconsistency in the temperature range annotations in the NCBI and GOLD databases, the four genomic regions were always identified as to possess GC content levels that are correlated with the temperature range conditions, suggesting their robustness to the possible annotation errors in the databases.

### Genomes whose temperature range conditions were consistently mis-classified

There were six genomes whose temperature range conditions were consistently mis-classified (in at least 80% of the cross validation experiments that they were used for testing), whether the prediction was based on the entire {*g_complete_*} or just the four genomic regions. The optimal growth temperature and the temperature range conditions of these six genomes are summarized in Table S-3 of the supplementary material. This result may prompt us to re-check the current temperature range condition annotations of the NCBI’s Microbial Genome Project Database. For example, we did find evidence to support our prediction for *Deinococcus geothermalis* DSM 11300 and did observe inconsistent annotations in the NCBI database for the temperature range conditions of *Methanococcus aeolicus* Nankai-3 and *Exiguobacterium sp.AT1b.Deinococcus geothermalis* DSM 11300 is labeled as mesophilic in the NCBI’s Microbial Genome Project Database, but is actually thermophilic with an optimum growth temperature of about 45°C to 50°C [48]. *Methanococcus aeolicus* Nankai-3 grows at temperatures between 20-55°C [49], and *Exiguobacterium* sp. AT1b grows at temperatures between 15-55°C [50]. The growth temperatures of both *Methanococcus aeolicus* Nankai-3 and *Exiguobacterium* sp. AT1b cross the boundaries of NCBI’s definition for mesophilic and thermophilic organisms and overlap more with the mesophilic category. However, *Methanococcus aeolicus* Nankai-3 is labeled as mesophilic while *Exiguobacterium* sp. AT1b is labeled as thermophilic in the NCBI’s Microbial Genome Project Database. These examples suggest that the correlation relationships between certain genomic and ecological traits of prokaryotic genomes can potentially facilitate “reverse ecology” [51], i.e., to fill/refine the environmental annotations of prokaryotic organisms based on their genomic features.

## Conclusions

We have investigated the correlation relationships between the GC content levels in the coding and surrounding non-coding regions of individual genes and the temperature range conditions of prokaryotic organisms based on the statistical tests and prediction tests. Through the KS tests conducted on all the mesophilic and thermo/hyperthermo-philic organisms, we have identified 413 genomic regions (277 coding and 136 non-coding regions) whose GC content levels show different distribution patterns for organisms under different temperature range conditions and that can be considered as potentially correlated with the temperature range condition. When these 413 genomic regions were used to predict the temperature range condition of an organism, the prediction accuracy can reach 92.45% for the complete genomes and 92.05% for the in-progress genomes. Four of these 413 genomic regions corresponding to the coding regions of K01251 (adenosylhomocysteinase), K03724 (DNA repair and recombination proteins), K07588 (LAO/AO transport system kinase), and K09122 (hypothetical protein) were consistently selected when the KS tests were conducted on partial lists of organisms with particular phylogenetic/oxygen requirement/habitat/salinity conditions being excluded and/or when the environmental annotations in the NCBI’s Microbial Genome Project Database or in the GOLD database were used. When these four genomic regions were used to predict the temperature range condition of an organism, the prediction accuracy reached 84.52% for the complete genomes, 84.09% for the in-progress genomes, and 82.70% for the metagenomes. Considering that they only account for less than 1% of all the 413 genomic regions potentially correlated with the temperature range condition but can to a great extent retain the prediction accuracy, we may interpret these four genomic regions as the core of the temperature range-correlated. Our results have also demonstrated that the correlation relationships between the genomic and ecological traits can potentially facilitate reverse ecology.

### Supplementary material

Supplementary materials for the conditions of different environmental factors, identified genomic regions, in-progress and meta-genomes, and consistently misclassified genomes are available at: http://users.ece.gatech.edu/~hzheng7/TempRanGC.pdf.

## Authors' contributions

HZ and HW have carried out the experiments and written the manuscript together.

## Competing interest

The authors declare that they have no competing interests.
